# 

**Published:** 1931

**Authors:** 


### The Medical Library of the University of Bristol,

WITH WHICH IS INCORPORATED

**The Library of the Bristol Medico-Chirurgical Society.**

*The following donations have been received since the publication of the list in June,* 1931.

October, 1931.

| | |
|---|---|
| British Medical Association (1) .. .. | 1 volume. |
| College of Physicians of Philadelphia (2) .. | 1 ,, |
| Mrs. E. S. Griffiths (3).. .. .. .. | 5 volumes. |
| Prof. E. W. Hey Groves (4) .. .. .. | 7 ,, |
| Mr. W. E. Marsh (5) .. .. .. .. | 20 ,, |
| Mayo Clinic and Mayo Foundation (6) .. | 1 volume. |
| Michell Clarke Memorial Fund (7) .. .. | 5 volumes. |
| Dr. George Parker (8) .. .. .. .. | 2 ,, |

Unbound periodicals have also been received from Professor E. W. Hey Groves.

### THE ONE HUNDRED AND THIRTY-SEVENTH LIST OF BOOKS.

The figures in round brackets refer to the figures after the names of the donors. The books to which no such figures are attached have either been bought from the Library Fund or received through the *Journal.*

| | | | | |
|---|---|---|---|---|
| **Abrams, A.** | *Spondylotherapy* | 6th Ed. | (5) | 1918 |
| **Baines, A. E.** | *Studies in Electro-Physiology* | | (3) | 1918 |
| **Bennet, N.** | *Science and Practice of Dental Surgery.* 2 vols. | 2nd Ed. | (7) | 1931 |
| **Boyd, M. F.** | *Preventive Medicine* | | (5) | 1925 |
| **Brewerton, T. Le G.** | *Treatise on the Mineral Water of Askern* | | | 1818 |
| **Brown, J. N. E.** | *Oliver Goldsmith, M.D., and his Medical Age* | | (8) | — |
| **Butler, G. R.** | *Diagnosis of Internal Medicine* | 4th Ed. | (5) | 1923 |
| **Campbell, H.** | *Aids to Pathology* | 4th Ed. | (5) | 1923 |
| **Clairmont, P., Winterstein, O., Dimtza, A.** | *Die Chirurgie der Tuberkulose* | | (4) | 1931 |
| | *Clinical Osteopathy* | | (5) | 1917 |
| **Davis, C. E.** | *Mineral Baths of Bath* | | | 1883 |
| **Firth, J. N.** | *Text-book of Chiropractic Symptomatology.* | 2nd Ed. | (5) | — |
| **Foster, A. L.** | *Principles and Practice of Chiropractic.* | 2nd Ed. | (5) | 1923 |
| **Goltra, J. N.** | *Preventive Medicine in the Home* | | (5) | — |
| **Kimber, D. C., and Gray, C. E.** | *Text-book of Anatomy and Physiology.* | 6th Ed. | (5) | 1924 |
| **Grover, B. B.** | *Handbook of Electrotherapy* | | (5) | 1924 |
| | *Handbook for Recently Qualified Practitioners* | | (1) | 1931 |
| **Hoffmann, V.** | *Verlauf der wichtigsten Knochen und Gelenkerkrankungen im Röntgenbilde* | | (4) | 1931 |
| **Joseph, J.** | *Nasenplastik and sonstige Gesichtsplastik nebst Mammaplastik* | | (4) | 1931 |
| **Konig, F.** | *Operative Chirurgie der Knockenbrüche.* Bd. 1 | | (4) | 1931 |
| **Lōhr, W., und Ratzfeld, L.** | *Die Bakteriologie der Wurmfortsatzentzündung und der appendikulären Peritonitis* | | (4) | 1931 |
| **McConnell, C. P., and Teall, C. C.** | *Practice of Osteopathy* | 4th Ed. | (5) | 1920 |
| **McCurdy, S. L.** | *Dissector in Abstract* | | (5) | — |
| **Martinet, A.** | *Clinical Diagnosis,* 2 vols. | 2nd Ed. | (5) | 1925 |
| | *Oeuvre, L', du Service de Sainté Militaire en Algérie,* 1830–1930 | | (4) | 1931 |
| **Orr, H. W.** | *Osteomyelitis and Compound Fractures* | | (4) | 1929 |
| **Pacini, A. J.** | *Outline of Ultra-Violet Ray Therapy.* | 2nd Ed. | (5) | 1923 |
| **Pottenger, F. M.** | *Symptoms of Visceral Disease* | 2nd Ed. | | 1923 |
| **Power, Sir D'Arcy** | *Selected Writings,* 1877–1930 | | (8) | 1931 |
| **Romanis and Mitchiner.** | *Science and Practice of Surgery* | 3rd Ed. | (7) | 1930 |
| **Rose, W. D.** | *Physical Diagnosis* | 3rd Ed. | (5) | 1922 |
| **Sainsbury, H.** | *Cardiac Cycle* | | | 1931 |

| | | | |
|---|---|---|---|
| **Schamberg, J. F.** | *Compend of Diseases of the Skin* 7th Ed. | (5) | — |
| **Slade, C. B.** | *Physical Examination and Diagnostic Anatomy* 3rd Ed. | (5) | 1923 |
| **Smith, F. J.** | *Lectures on Medical Jurisprudence and Toxicology* | (3) | 1900 |
| **Tanner, T. H.** | *Practice of Medicine*, 2 vols. | (3) | 1875 |
| *Truth About Cancer* | | (7) | — |
| **Wallgren, A. B.** | *Pathology in Abstract* 3rd Ed. | (5) | — |
| **Wanklyn, J. A., and Chapman, E. T.** | *Water Analysis* 8th Ed. | (3) | 1891 |

TRANSACTIONS, REPORTS, JOURNALS, ETC.

| | | |
|---|---|---|
| College of Physicians of Philadelphia, Transactions. 3rd Series. Vol. 52. 1930 | (2) | 1930 |
| Mayo Clinic and Mayo Foundation. Collected papers of. Vol. xxii. 1930 | (6) | 1930 |
| Medical Society's Transactions. Vol. liv. | | 1931 |
| Progressive Medicine, June, 1931 | | 1931 |
| Surgical Clinics of North America. June, 1931 | | 1931 |

### Local Medical Notes

EXAMINATION RESULTS.

**University of Bristol.**—Students of the University have recently passed the following examinations :—

M.B., CH.B.—*First Examination. Pass :* R. D. Cæsar, Monica L. Hawkins, E. J. Lace, J. S. W. Little, Coralie W. Rendle-Short, L. A. Schnipelsky, A. M. Spencer, E. Want, E. L. Ward, P. Zimmering. *In Botany (Completing Requirements) :* V. T. Baxter, J. G. Field, A. W. Woolley.

B.D.S.—*First Examination. Pass : In Botany (Completing Requirements) :* J. W. E. Snawdon.

L.D.S.—*Preliminary Science Examination. Pass : In Biology (Completing Examination) :* R. E. Buchan, Frances M. Orton.

**Conjoint Board.**—M.R.C.S., L.R.C.P.—*In Chemistry :* J. T. S. Hutchins, B. E. Ffrench. *In Physics :* J. T. S. Hutchins, Mabel L. Glenny, Gwyneth M. Griffiths. *In Elementary Biology :* B. E. Ffrench, Mabel L. Glenny, Gwyneth M. Griffiths, J. A. Cochrane, P. W. M. Martin. *In Anatomy :* L. E. Sealy, C. F. Rivington. *In Physiology :* C. F. Rivington, T. H. Taylor. *In Pharmacology and Materia*

*Medica :* R. H. Moodie. *In Pathology :* J. J. Kempton, G. M. Evans. *In Medicine :* L. C. Holland.* *In Surgery :* D. Johnson.* *In Midwifery :* A. M. Davies, Enid A. Taylour, J. J. Kempton.

L.D.S., R.C.S.—*Part II. Pass :* T. A. A. Eaves, W. U. Harwood.

---

**Long Fox Memorial Lecture.**—The Lecture for 1931 will be delivered on 27th October at the University at 5.0 p.m. by Mr. A. L. Flemming, who will take as his subject " The Partnership between Anæsthesia and Surgery."

The British Association of Dermatology and Syphilology held its Annual Meeting in Bristol on 2nd and 3rd July, under the Presidency of Dr. Kenneth Wills. The subjects under discussion comprised : " Pityriasis Rosea," introduced by Dr. Sydney Thompson and Dr. G. H. Percival ; " Hyperglycæmia in Skin Diseases," by Professor Rost of Freiburg ; " Erythema Nodosum," by Dr. J. O. Symes ; " Gastric Secretion in Psoriasis Vulgaris," by Dr. W. Dyson ; " Lichen Urticatus," by Dr. Rupert Hallam ; " A survey of modern development in Leprosy work, with an appreciation of the present position," by Dr. Robert Cochrane ; " Observations on the deposit of Gold in the skin," by Dr. Wigley ; " Autohæmotherapy," by Dr. Norman Burgess. The meetings were well attended, and the Annual Dinner was held at the Victoria Rooms on 2nd July, and various excursions were carried out.

* Qualified.

### Publications Received

From Adlard & Son Ltd.:

*A Pocket Atlas and Text-book of the Fundus Oculi.* 2nd Edition. By G. L. Johnson, M.A., B.C., M.D., F.R.C.S. Price 12s. 6d.

From Baillière, Tindall & Cox.

*A Catalogue of the Publications of Baillière, Tindall & Cox in Medicine and Science.* June, 1931.

From Cambridge University Press:

*Epidemiology and Control of Malaria in Palestine.* By Israel J. Kligler. Price $5.00. Published by the University of Chicago Press.

From William Heinemann (Medical Books) Ltd.:

*The Thyroid and Manganese Treatment.* By H. W. Nott, M.R.C.S., L.R.C.P. Price 7s. 6d.

*Practical Morbid Histology.* 2nd Edition. By Robert Donaldson, M.A., M.D., Ch.B., F.R.C.S.E., D.P.H. Price 42s.

From E. Hopewell-Ash:

*An A.B.C. of Medical Hypnosis.* By E. Hopewell-Ash, M.D., B.S., M.R.C.S., L.R.C.P. Price 2s.

From The Lancet Ltd.

*Motives and Mechanism of the Mind.* By E. Graham Howe, M.B., B.S., D.P.M. Price 10s. 6d.

From H. K. Lewis & Co. Ltd.:

*Respice-Prospice.* Lewis's, 1844–1931.

*Gould's Medical Dictionary.* 3rd Edition. Price 30s.

*Fundamental Principles of Ray Therapy.* By William Beaumont, M.R.C.S., L.R.C.P. Price 6s.

*Notes on Radium Therapy for Medical Students.* By Hector A. Colwell, M.B., Ph.D., M.R.C.P., D.P.H. Price 6s.

From Oxford University Press:

*Applied Physiology.* 4th Edition. 1931. By Samson Wright, M.D., M.R.C.P. Price 18s.

*Encephalitis Lethargica.* By Constantin von Economo. Price 18s.

*The Commoner Nervous Diseases.* By F. J. Nattrass, M.D., F.R.C.P. Price 12s. 6d.

From Straker Bros. Ltd.:

*Hay Fever, Hay Asthma.* By William Lloyd, F.R.C.S.

From John Wright & Sons Ltd.:

*Demonstrations of Physical Signs in Clinical Surgery.* By Hamilton Bailey, F.R.C.S. Price 21s.

*Studies in the Photo-activity and Therapy of the Tungsten-titanium Arc.* By J. Burdon-Cooper, M.D., B.S., B.Sc., F.R.C.S.E., D.O., F.C.S., and Arthur Roberts, T.D., F.R.C.S.E., M.R.C.S. Price 10s.

*"First Aid" to the Injured and Sick.* By Warwick and Tunstall. 13th Edition. Price 2s. 6d.

